# Development of surface engineered antigenic exosomes as vaccines for respiratory syncytial virus

**DOI:** 10.1038/s41598-021-00765-x

**Published:** 2021-11-01

**Authors:** Suyeon Hong, Shaobo Ruan, Zachary Greenberg, Mei He, Jodi L. McGill

**Affiliations:** 1 Department of Veterinary Microbiology and Preventive Medicine, Iowa State University, Ames, IA USA; 2 Department of Pharmaceutics, College of Pharmacy, University of Florida, Gainesville, FL USA

**Keywords:** Microfluidics, Peptide vaccines, Peptide delivery

## Abstract

Respiratory syncytial virus (RSV) is one of the main pathogens associated with lower respiratory tract infections in infants and young children worldwide. Exosomes secreted by antigen presenting cells (APCs) can elicit immune responses by carrying major histocompatibility complex (MHC) class I molecules complexed with antigenic peptides and other co-stimulating factors. Therefore, we developed novel immunomagnetic nanographene particles to sequentially isolate, surface engineer, and release intact dendritic cell (DC) exosomes for use as a potential vaccine platform against RSV. The H-2D^b^-restricted, immunodominant peptides from RSV (M_187–195_ and NS1_61–75_) were introduced to MHC-I on DC-derived exosomes to express peptide/MHC-I (pMHC-I) complexes. A mouse model of RSV infection was used to define the immunogenicity of surface engineered exosomes for activating virus-specific immune responses. Ex vivo assays demonstrated that engineered exosomes carrying RSV-specific peptides can elicit interferon-gamma (IFN-γ) production by virus-specific CD8+ T cells isolated from RSV-infected C57BL/6 mice. In vivo assays demonstrated that subcutaneous administration of both M_187–195_ and NS1_61–75_ engineered exosomes to mice, with or without additional adjuvant, appeared safe and well tolerated, however, did not prime antigen-specific CD8+ T cell responses. Surface engineered exosomes are immunogenic and promising for further development as a vaccine platform.

## Introduction

Respiratory syncytial virus (RSV) is one of the main causes of severe lower respiratory tract infections in infants and young children. The incidence of RSV-associated acute lower respiratory infection has been estimated approximately 33.1 million in 2015 globally, with total deaths more than 118,000 in children younger than age 5^1^. Despite years of research, there are currently no available licensed vaccines against human RSV infection. Hence, it is important to develop effective vaccines to prevent such viral infections and control RSV-induced disease.

Exosomes are small (30–150 nm) extracellular vesicles (EVs) secreted from living cells and involved in intercellular communication by containing various signaling molecules such as lipids, proteins, DNAs, and RNAs^2,3^. Several studies have reported that exosomes secreted from immune cells are immunogenic and can transport molecules over long distances for eliciting immune responses^4^. For example, exosomes released by B cells carry major histocompatibility complex (MHC) class II on their surface and have the capacity to stimulate T cells^5^. Human natural killer (NK) cell-derived exosomes expressing NK cell markers exhibit cytotoxicity against tumor cells^6^. Exosomes from *Mycobacterium bovis* Bacillus Calmette–Guérin (BCG)-infected macrophages also have the capacity to stimulate CD4+ and CD8+ T cells from BCG-infected mice in vitro, and to promote naïve CD4+ and CD8+ T cell responses in vivo^7^. Particularly, exosomes released from antigen presenting cells (APCs) such as dendritic cells (DCs) have emerged as potential immunotherapy due to their stability during storage, composition of immune regulatory molecules, high biocompatibility, and safety^8,9^. Immature DCs maintain T cell tolerance, whereas mature DCs have a higher number of peptide/MHC (pMHC) complexes and costimulatory molecules such as CD40 and CD80/86 interacting with CD40 ligand and CD28, respectively, on the T cell, rendering them highly effective at inducing T cell responses^10^. Herein, DC-derived exosomes can be taken up and subsequently processed by APCs or present pMHC complexes to elicit antigen-specific, MHC-restricted, T cell responses^11^. For instance, exosomes derived from DCs of male mice with functional pMHC complexes are able to elicit responses from naïve, transgenic, male-antigen-specific CD4+ T cells both in vivo and in vitro^12^. It also has been reported that exosomes secreted by virus-infected cells can elicit immune responses due to carrying viral-specific proteins and RNAs^13^. Montaner-Tarbes et al*.* demonstrated that pigs vaccinated with serum-derived exosomes isolated from pigs previously infected with porcine respiratory and reproductive virus had the capacity to elicit virus-specific humoral immune responses^14^. Exosomes carrying the spike S protein from SARS-CoV have also been recently shown to elicit SARS-CoV-specific neutralizing antibody titers^15^. Due to the innate immunogenicity and safety and evident importance in cell signaling in vivo, an exosome-based vaccine has gained significant interest in aspects of research and clinical application.

Due to the capacity for DC-derived exosomes to elicit robust adaptive immune responses, we previously developed a microfluidic-based cell culture chip to enable real-time isolation and engineering of exosomes secreted from DCs with streamlined systems^16^. This showed high efficiency in generating exosomes harboring pMHC complexes, which induced in vitro proliferation of transgenic, gp100-specific CD8+ T cells in the presence of APCs^16^. Here, we further extended the work by investigating the potential for developing an engineered, antigenic exosome-based vaccine against a common viral infection. In this work, we further developed novel immunomagnetic nanographene particles (ExoRelease) with the function of immunocapture and on demand light-triggered release, which is suited for large scale exosome isolation and surface engineering. The streamlined workflow and simple operation for surface engineering of therapeutic peptides on DC exosomes have not been reported elsewhere. Due to the formation of pMHC-I complexes on exosome surface, this platform is amendable for highly efficient production of therapeutic peptide-tailored exosome vaccines. Exosomes were surface-engineered to harbor two well-described, immunodominant, H-2D^b^-restricted peptides from RSV: the M_187–195_ and NS1_61–75_ peptides^17,18^. Their potency and immunogenicity were investigated, and we observed robust interferon-gamma (IFN-γ) production by activated RSV-specific CD8+ T cells ex vivo. Using the mouse model with RSV infection, we observed safe administration without any adverse effects, however, vaccination with surface-engineered exosomes did not elicit antigen-specific CD8+ T cell responses in vivo. Thus, our results show that surface engineered exosomes have the capacity to activate RSV-specific T cell responses, but further research is needed to validate their immunogenicity in vivo.

## Results

### Harvest and characterize RSV specific peptide engineered DC exosomes

**Figure 1:**
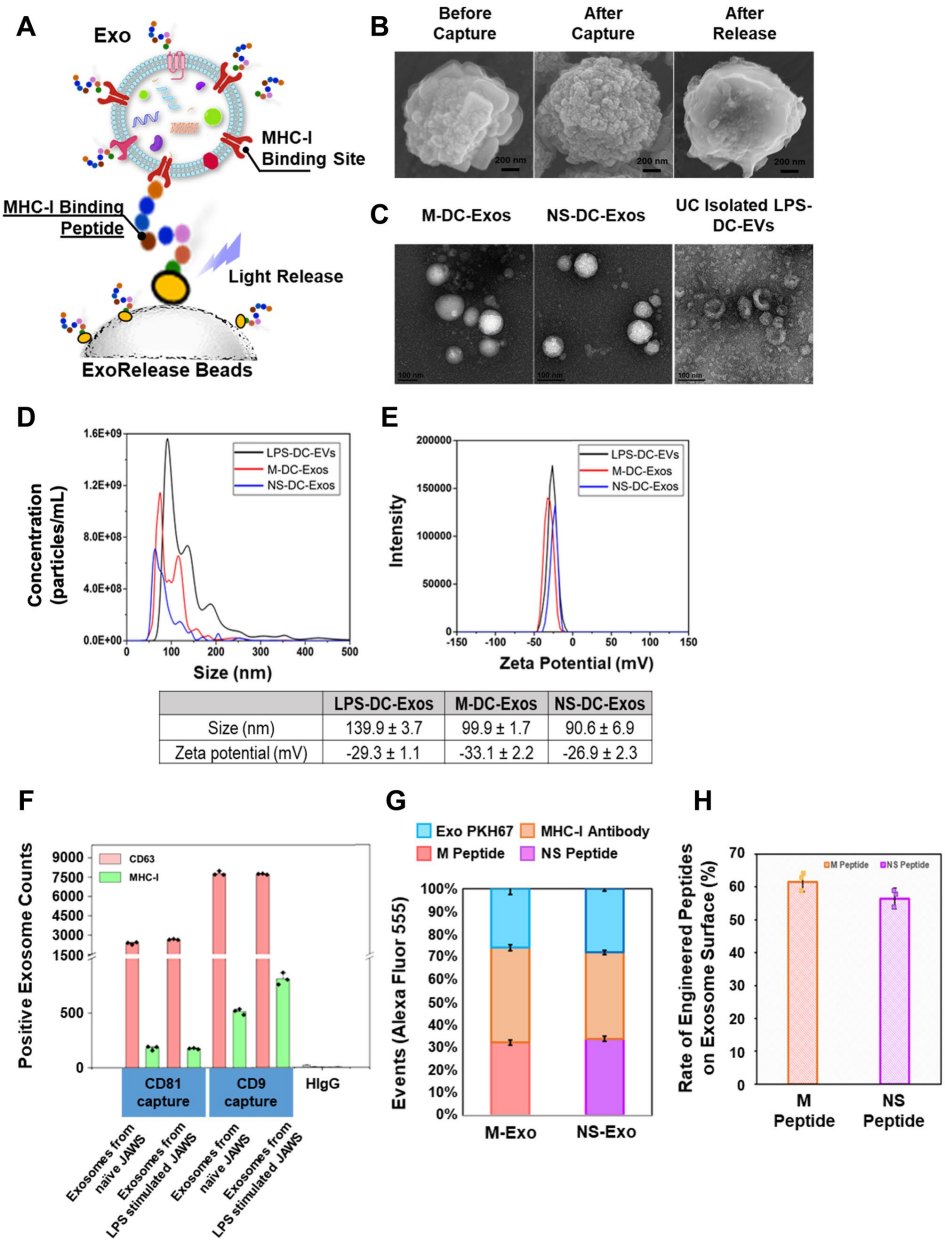
Characterization of RSV-Specific Peptide Engineered DC Exosomes. (**A**) Schematic illustration of ExoRelease beads prepared, peptide-engineered exosomes via MHC binding peptide capture, surface MHC peptide coating, and photo-release for harvesting. (**B**) SEM images showing the ExoRelease bead surface morphology during the exosome capture, surface engineering, and release of intact captured exosomes. The entire surface was covered by round-shape exosomes after capture which is significantly different than the bare surface after release of intact captured exosomes. (**C**) TEM images showing the morphology of harvested exosomes using ExoRelease beads approach, which is in uniform size and round shape. However, ultracentrifugation isolated vesicles are more heterogeneous with substantial particle aggregates. (**D**) The nanoparticle tracking analysis of harvested, peptide-engineered exosomes using ExoRelease bead approach, compared with ultracentrifugation isolated vesicles without peptide engineering. Here two peptides M and NS were prepared for exosome surface engineering. The peptide engineered exosomes are in much narrower size distribution. (**E**) Zeta potential analysis of surface engineered exosomes compared with ultracentrifugation isolated vesicles as the control group. The zeta potential from peptide engineered exosomes were slightly changed due to the surface engineering, but still maintain the good integrity. (**F**) NanoView microarray was used to determine the level of MHC-I the exosomes. Exosomes from LPS stimulated JAWS expressed more MHC-I on their surface. (**G**) The stacked bar chart showing the percentage distribution of total exosomes, total MHC-I on exosome surface, and total RSV peptides (M and NS) bound to exosome MHC-I, which is analyzed by bead-based flow cytometry. (**H**) The rate of engineered RSV peptides (M and NS) on exosome surfaces (n = 3, RSD < 5%).

In our previous work, we have shown that polydimethylsiloxane (PDMS) polymer made microfluidic culture chip engineered tumor antigenic DC exosomes possessed the ability to activate transgenic, antigen-specific CD8+ T cells^16^. Herein, we further developed a novel immunomagnetic nanographene particles (ExoRelease) with the function of immunocapture and on demand light-triggered release (Fig. 1A). The RSV-specific peptides (M_187–195_ or NS1_61–75_) can form the binding complex with MHC-I binding sites on the exosome surface. Particularly, DC-derived exosomes contain highly abundant MHC-I binding sites for antigenic presentation and immune activation. Here we used mature JAWS cell-derived exosomes stimulated by LPS (Lipopolysaccharide from *E. coli* 0111:B4). Therefore, MHC-I positive exosomes can be captured by such peptides conjugated with a photo-cleavage linker on the surface of ExoRelease particles. The unique nano-morphology of these ExoRelease particles was shown in Fig. 1B, which enables more efficient capture of exosomes than conventional beads due to much larger surface area and comparable nano-scale cavities^19^. After capture, the uniform, dense, and nano-sized exosomes were shown covering the entire surface of ExoRelease particles (Fig. 1B). Subsequently, the peptides either M_187–195_ or NS1_61–75_ can be introduced to incubate with captured exosomes for surface engineering. The intact, engineered exosomes can be harvested via the light-triggered release. Figure 1B SEM images showed the morphology of ExoRelease particles after photo-release of intact engineered exosomes, which is clean and comparable with the morphology before capture, indicating the effective exosome isolation, surface engineering, and photo-release. TEM images of harvested engineered exosomes harboring either M or NS1 peptides were shown in Fig. 1C. The uniform, round-shape vesicles in a size range around 100 nm indicate the good quality of harvested exosomes. We did not observe any morphology differences between M_187–195_ peptide and NS1_61–75_ peptide engineered exosomes. The harvested engineered exosomes showed much purer quality than the exosomes isolated from ultracentrifugation approach which displayed cell debris and aggregates (Fig. 1C). According to the nanoparticle tracking analysis, the non-engineered, LPS stimulated DC exosomes isolated by ultracentrifugation displayed a size distribution around 139.9 ± 3.7 nm shown in Fig. 1D. In contrast, both M_187–195_ and NS1_61–75_ peptide engineered exosomes exhibited narrower size distribution around 90–100 nm (Fig. 1D). The zeta potential is an important and readily measurable indicator of surface properties of EVs and exosomes, which can assess the influences from surface chemistry and bioconjugation applied^20^. In Fig. 1E, we observed a slight reduction from M_187–195_ peptide engineered exosomes and a slight increase from NS1_61–75_ peptide engineered exosomes. Due to the positive charge property of M_187–195_ peptide and the negative charge property of NS1_61–75_ peptide, such zeta potential changes indicate the successful surface engineering of DC exosomes with RSV-specific peptides (Fig. 1E). We also used the single-EV microarray imaging technology from NanoView to directly determine the MHC-I expression level from prepared exosomes as well as their functional markers shown in Fig. 1F. The specific antibody capture on each NanoView chip spot allows the affinity capture of exosomes based on their surface markers for further multiplexed affinity probing. Here we used CD63 and MHC-I antibodies for probing. Multiplexed fluorescence images of each antibody capture spot can derive the total counts of positive exosomes expressing MHC-I. The isotype spots (HIgG) were used as the background and non-specific control. We observed that CD9 positive exosome capture spots displayed good expression level of MHC-I, other than CD81 capture spots, which indicates that JAWS exosomes are mainly CD9 positive and LPS stimulated JAWS exosomes express more MHC-I on the surface. The CD63 is the common structural and functional proteins during exosome biogenesis, which serves as the exosome reference marker to assess the total exosomes. Our observation in Fig. 1F displayed comparable abundance between all samples and chip spots, indicating the consistent and reproducible preparation of JAWS exosomes for peptide engineering. As seen in Fig. 1G and H, we used fluorescence labeling and CytoFlex flow cytometry to identify the percentage of total MHC-I on the exosome surface, as well as the percentage of total peptide bound to exosome MHC-I. By normalization, we could get the rate of engineered peptides on the exosome surface, which is about ~ 60%. Thus, the RSV peptides were sufficiently engineered on the exosome surface.

### RSV specific peptide engineered DC exosomes activate RSV specific CD8 T cells ex vivo

**Figure 2:**
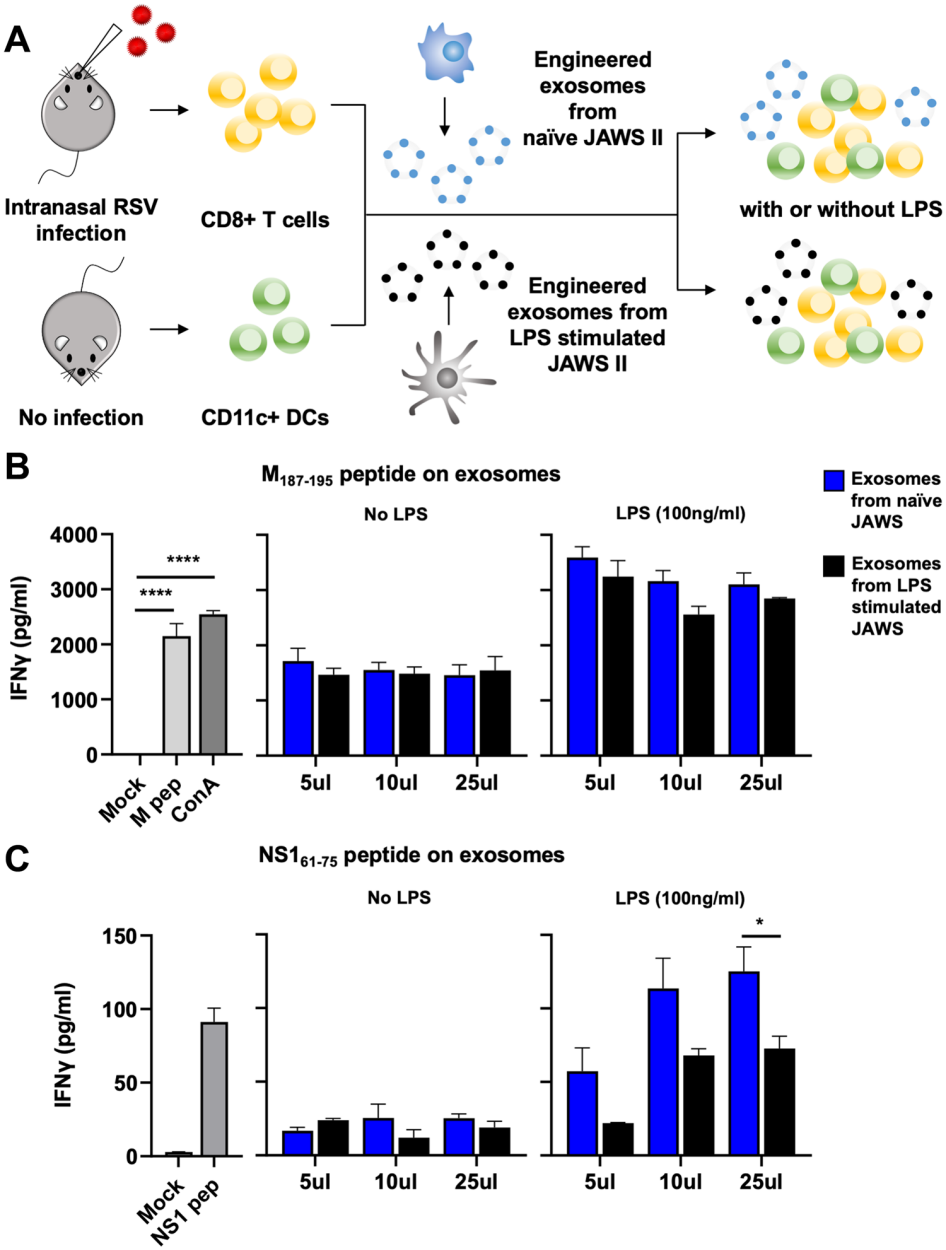
Engineered exosomes were immunogenic for activating RSV-specific CD8 T cells ex vivo. (**A**) Schematic illustration of the establishment of a DC-CD8 T cell co-culture model. C57BL/6J mice were infected intranasally with RSV strain A2 to generate RSV-specific CD8+ T cells. Magnetic-activated cell sorting (MACS) was used to sort CD8+ T cells from RSV-infected mice and CD11c + DCs from non-infected mice. CD8+ T cells and CD11c + DCs (5:3 ratio) were incubated for 72 h with escalating concentrations (5 μL, 10 μL, 25 μL) of exosomes engineered with M_187–195_ or NS1_61–75_ peptides with or without LPS (100 ng/mL). (**B**, **C**) Cell culture supernatants were collected on day 3 of stimulation with M_187–195_ or NS1_61–75_ engineered exosomes. The samples were diluted 1:4 and analyzed by commercial ELISA for IFN-γ. Data represent means ± SEM. ****p < 0.0001 as determined by one-way ANOVA and Dunnett's multiple comparisons test. *p < 0.05 as determined by two-way ANOVA and Šídák's multiple comparisons test.

To determine if engineered exosomes have the capacity to elicit virus-specific immune responses ex vivo, we first established a CD8 T cell-APC co-culture model (Fig. 2A). To generate RSV-specific CD8+ T cells, C57BL/6J mice were infected intranasally with RSV strain A2. On day 8 after infection, the mice were sacrificed and CD8+ T cells were isolated from the lungs and spleen by negative selection using magnetic cell separation. In our prior study, we determined that non-engineered or peptide-engineered exosomes alone are not sufficient to induce in vitro CD8 T cell activation, and the presence of an APC is required^16^. Therefore, CD11c + DCs were isolated from naïve C57BL/6J mice to serve as APC. Purified CD8+ T cells were mixed in a 5:3 ratio with naïve CD11c + dendritic cells. Engineered exosomes loaded with either RSV M_187–195_ (NAITNAKII) or NS1_61–75_ (ICPNNNIVV) peptides were added in escalating concentrations to the cell cultures. It has been previously demonstrated that exosomes secreted from activated DCs carry costimulatory molecules such as CD80, CD86 and CD40, which are necessary for activating antigen-specific CD8+ T cells^10,21^. Therefore, we hypothesized that exosomes from matured DCs could be more efficient for activating CD8+ T cells, compared to exosomes from naïve DCs. To this end, T cell:DC cultures were stimulated with engineered exosomes derived from either LPS-stimulated or naïve DCs (JAWS II cell line). On day 3 of stimulation, cell culture supernatants were collected and analyzed for IFN-γ by ELISA. As seen in Fig. 2B (the 2nd panel), M_187–195_ peptide engineered exosomes derived from both immature and mature DCs induced antigen-specific IFN-γ production by the CD8+ T cells, with no significant differences in the response to the different types of exosomes. However, with the presence of mature DCs in the co-culture, the IFN-γ production by the CD8+ T cells was about twofold higher than that with the presence of naïve DCs (Fig. 2B, the 3rd panel). A similar trend was observed with the NS1_61–75_ peptide engineered exosomes (Fig. 2C), in which exosomes from either naïve or LPS-activated JAWSII DC cells elicited antigen-specific IFN-γ production by the CD8+ T cells. However, the co-cultured mature DCs played an essential role for facilitating CD8+ T cell activation as indicated by the significantly increased production of IFN-γ in the stimulated cell culture supernatants. Control wells for the T cell:DC co-cultures are shown in Fig. 2B and C (the 1st panels). Compared to mock-stimulated controls, stimulation with the M_187–195_ or NS1_61–75_ peptide only, as well as the nonspecific mitogen ConA, induced robust, antigen-specific IFN-γ. Interestingly, the concentrations of IFN-γ induced by the engineered-exosomes was higher than that from the peptide stimulated control cultures, suggesting that the surface-engineered exosomes are very efficient activators of CD8+ T cell responses ex vivo, and could be highly immunogenic for activating RSV-specific CD8+ T cell responses in vivo.

### Safety and immunogenicity of RSV specific peptide engineered DC exosomes in vivo

**Figure 3:**
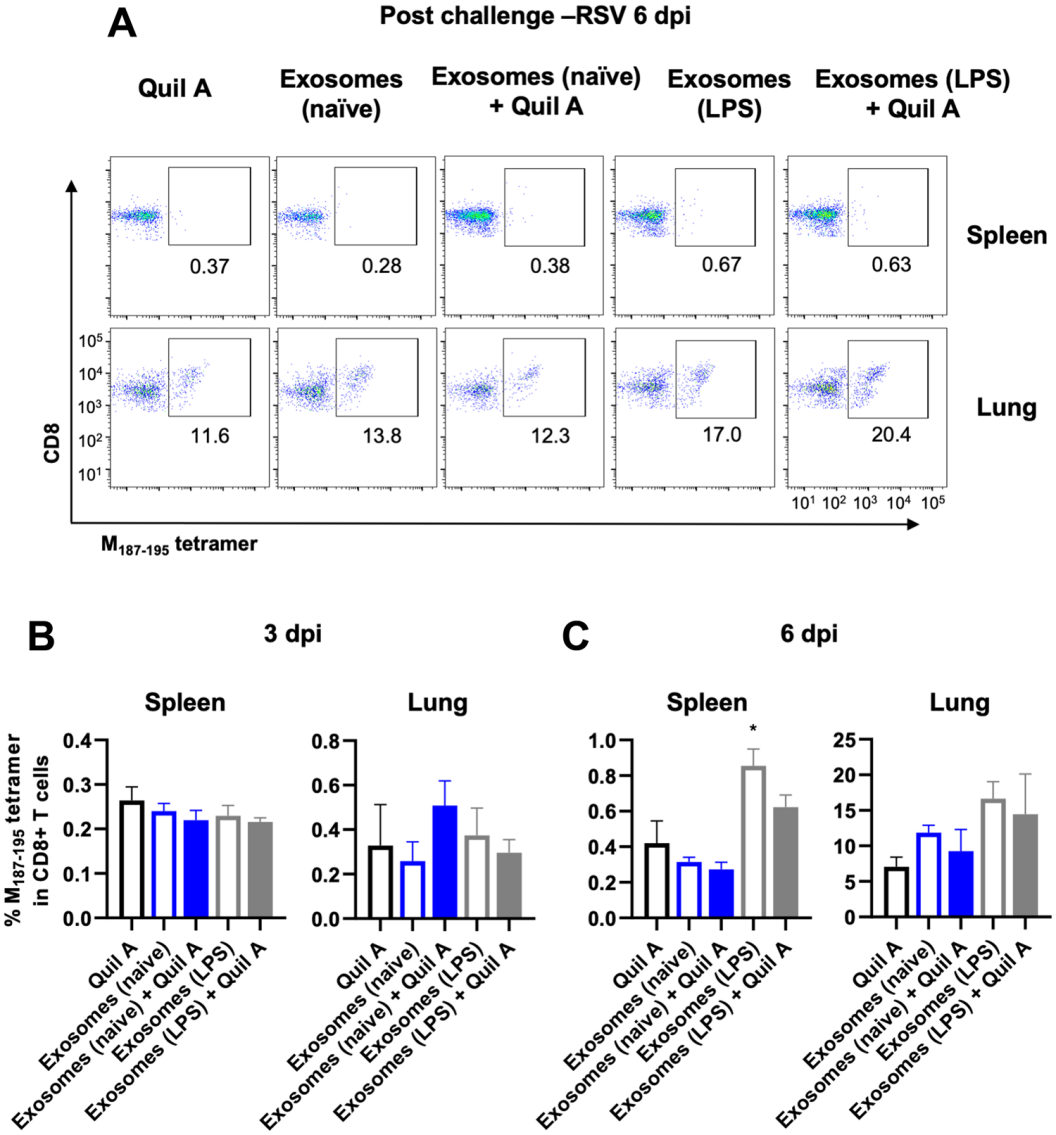
Confirmation of M_187–195_-specific CD8 T cells by tetramer staining. Groups of vaccinated mice were sacrificed either 3 or 6 days post-infection (dpi). Their lungs and spleens were stained with antibodies and analyzed by flow cytometry. (**A**) Representative flow cytometry plots of lungs and spleens at 6 dpi of RSV. (**B**, **C**) The antigen-specific CD8 T cells were determined based on M_187–195_-tetramer staining. Data are presented as means ± SEM. *p < 0.05 as determined by one-way ANOVA and Holm-Šídák's multiple comparisons test.

**Figure 4:**
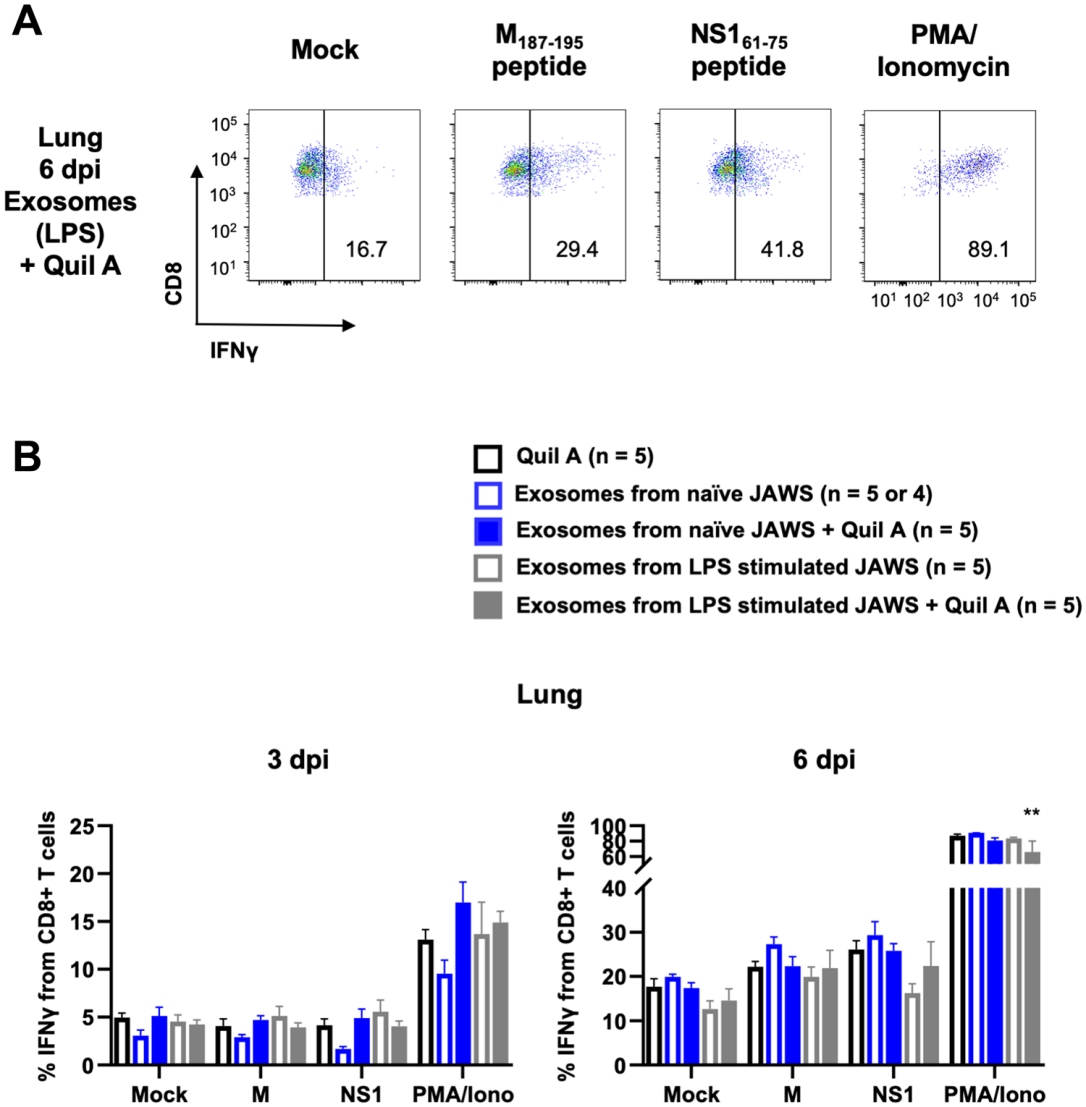
The production of IFN-γ by M_187–195_ or NS1_61–75_-specific T cells in the lungs. Groups of vaccinated mice were sacrificed either 3 or 6 days post-infection (dpi). Their lungs were stained with antibodies and analyzed by flow cytometry. (**A**) Representative flow cytometry plots showing IFN-γ producing lungs from a group of exosomes from LPS stimulated JAWS with Quil A at 6 dpi of RSV after stimulation with M_187–195_ or NS1_61–75_ peptides. (**B**) Flow cytometric analysis of IFN-γ production by lungs in the CD45+ CD8+ T cell population after stimulation with the indicated conditions. Data are presented as means ± SEM. **p < 0.01 as determined by two-way ANOVA and Tukey's multiple comparisons test.

We next determined the safety and immunogenicity of RSV-specific peptide engineered DC exosomes in the C57BL/6 mouse model. In our initial studies, groups of mice were vaccinated subcutaneously with a mixture of both types of peptide-bearing exosomes (M_187–195_ and NS1_61–75_) from LPS-matured JAWSII DC cells. Mice were vaccinated with engineered exosomes only, or with engineered exosomes plus a Quil A adjuvant. Second and third booster immunizations were performed at 3-week intervals. No adverse reactions or vaccine-site reactions were observed at any time in the mice, suggesting that the engineered-exosomes are safe and well tolerated. The mice were sacrificed 3 weeks following the final immunization and assessed for antigen-specific CD8 + T cell responses in the spleen and lung. No M_187–195_ or NS1_61–75_-specific CD8 + T cell responses were detected in these animals (data not shown). It is possible that exosome-immunization primed antigen-specific CD8 T cells, but that the responses were too low to be detected. However, an anamnestic increase in the virus-specific T cell response may be detectable following a viral challenge. To address this hypothesis, we studied five groups of animals by vaccinating subcutaneously with Quil A (10 μg/mouse), a mixture of exosomes (M_187–195_ and NS1_61–75_) from naïve JAWS cells (100 μL/mouse), a mixture of exosomes (M_187–195_ and NS1_61–75_) from naïve JAWS cells (100 μL/mouse) with Quil A, a mixture of exosomes (M_187–195_ and NS1_61–75_) from LPS stimulated JAWS cells (100 μL/mouse), or a mixture of exosomes (M_187–195_ and NS1_61–75_) from LPS stimulated JAWS cells (100 μL/mouse) with Quil A, respectively. A booster immunization was performed two weeks later and then mice were challenged via intranasal inoculation with 10^7^ plaque-forming unit (PFU) of RSV strain A2. Groups of mice were sacrificed either 3 or 6 days post-infection (dpi) and their lungs and spleens were analyzed for M_187–195_-specific CD8 T cells by tetramer staining (Fig. 3) or for both M_187–195_ and NS1_61–75_-specific T cells by intracellular cytokine staining (Fig. 4). Representative M_187–195_ tetramer staining from 6 dpi is shown in Fig. 3A. There were no significant differences in the frequency of M_187–195_-specific CD8 T cells among groups in the lung or spleen at 3 dpi, and the frequency of M_187–195_-specific CD8 T cells was not above background for any group, suggesting that there were no anamnestic CD8 T cell responses induced by exosome vaccination and challenge (Fig. 3B). In contrast, by day 6 post-infection, significant numbers of tetramer-positive CD8 T cells were detected in both the lungs and spleen of all groups (Fig. 3C). However, there were no differences in the frequency of cells detected in vaccinated vs. non-vaccinated animals.

**Figure 5:**
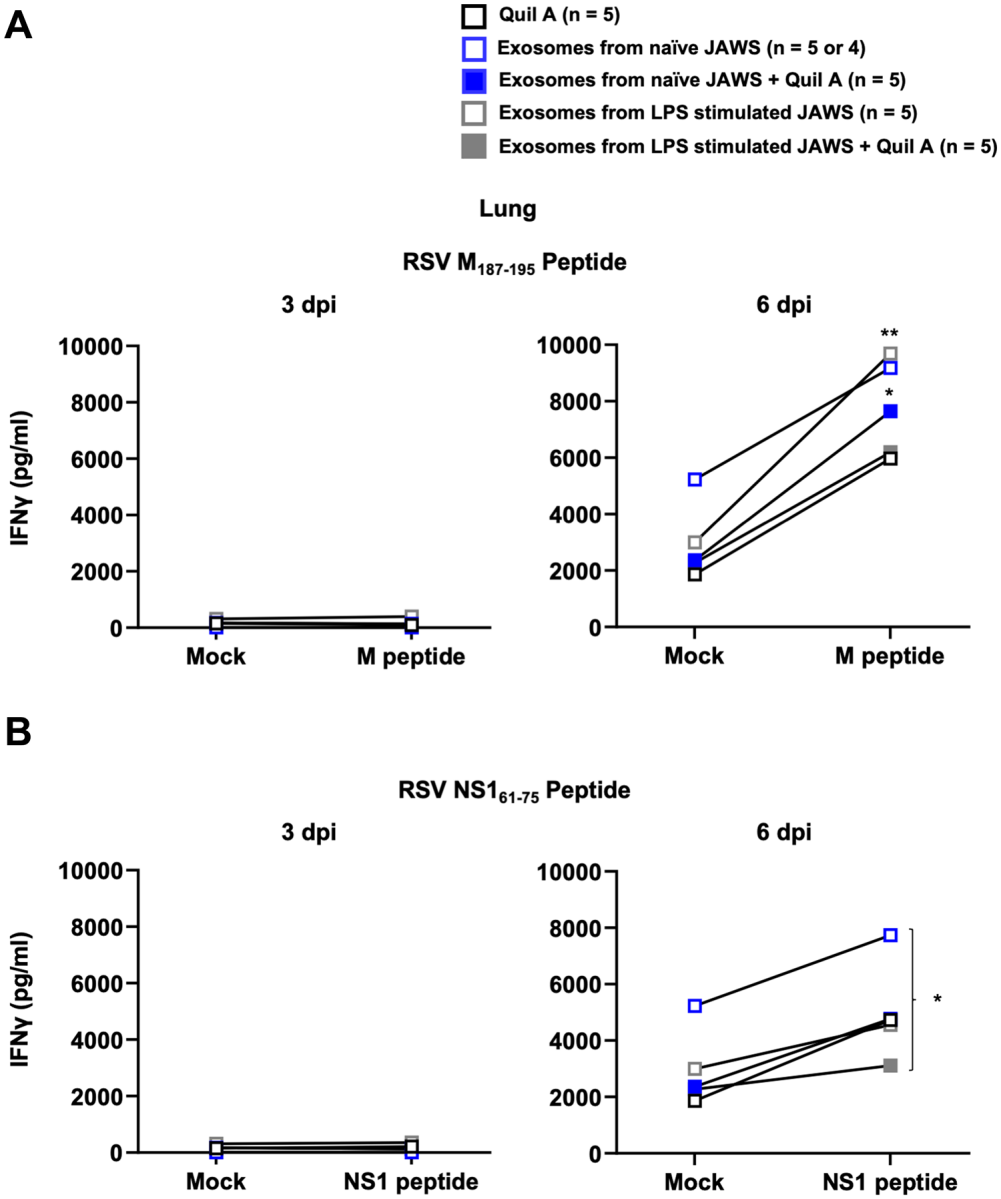
Vaccination with the surface engineered exosomes was not able to elicit exosome-induced memory T cell responses in vivo*.* Groups of vaccinated mice were sacrificed either 3 or 6 days post-infection (dpi). Cells from the lungs were stimulated with (**A**) M_187–195_ or (**B**) NS1_61–75_ peptides for 72 h. An ELISA analysis for IFN-γ was performed on the cell culture supernatants. Data are presented as means ± SEM. (**A**) *p < 0.05, **p < 0.01 as determined by two-way ANOVA and Šídák's multiple comparisons test. (**B**) *p < 0.05 as determined by two-way ANOVA and Tukey's multiple comparisons test.

Functional T cell responses were also measured by intracellular cytokine staining (ICS) and ELISA for IFN-γ using antigen-recall assays. Figure 4A shows representative flow cytometry data from the lungs of a mouse that was vaccinated with exosomes from LPS-matured JAWS cells plus Quil A on 6 dpi. The frequency of CD8 T cells producing IFN-γ in response to stimulation with the M_187–195_ or NS1_61–75_ peptides was not above background on day 3 post-infection for any group, but was detectable in all groups by day 6 (Fig. 4B). Cells from the lungs of vaccinated and infected mice were also stimulated for 72 h and an ELISA analysis for IFN-γ was performed on the cell culture supernatants. Consistent with our flow cytometry results, we observed no M_187–195_ or NS1_61–75_-specific IFN-γ production from any group on 3 dpi (Fig. 5). On 6 dpi, M_187–195_ and NS1_61–75_-specific IFN-γ production was detected from all groups of animals, consistent with the response to RSV challenge (Fig. 5). Lung cells collected from mice vaccinated with exosomes from naïve JAWS + Quil A (blue filled box, 3rd group) or from LPS stimulated JAWS (white filled & gray line box, 4th group) showed significant M_187–195_-specific IFN-γ production (Fig. 5A). In Fig. 5B, we noted a significant difference in NS1_61–75_-specific IFN-γ production between the lung cells from mice vaccinated with exosomes from naïve JAWS (white filled & blue line box, 2nd group) and those with exosomes from LPS stimulated JAWS + Quil A (gray filled box, 5th group) (Fig. 5B). However, we observed no other differences between treatment groups. Taken together, these results suggest that RSV peptide surface-engineered exosomes are highly immunogenic ex vivo; however, vaccination with the MHC-I surface-engineered M_187–195_ and NS1_61–75_ exosomes was not able to elicit exosome-induced memory T cell responses in vivo.

## Discussion

RSV is the major cause of severe lower respiratory tract infections in infants and children worldwide, resulting in high number of hospitalization, morbidity, and mortality^22,23^. There is currently no vaccine available to prevent RSV infection. In this project, we aimed at developing peptide-engineered exosomes as a potential vaccine platform to elicit CD8 T cell responses against RSV. Here, we demonstrated that the novel, immunomagnetic ExoRelease particle method is effective to surface engineer DC-derived exosomes with the immunodominant H-2D^b^-restricted M_187–195_ or NS1_61–75_ peptides from RSV. The NTA analysis and Zeta potential characterization, as well as the TEM and SEM morphological study well support that we can successfully load RSV-specific peptides on the surface of DC-derived exosomes. The isolated exosomes are in a narrow and uniform size range around 100 nm with high integrity. Compared to currently existing methods, our ExoRelease particle method is streamlined with simple preparation protocols, which is amendable for highly efficient, large-scale production of therapeutic peptide-tailored exosome vaccines.

Our results demonstrate that DC-derived RSV-peptide engineered exosomes activate CD8 T cells ex vivo when cultured in the presence of DCs. We have previously observed that culturing non-engineered or peptide engineered-exosomes alone in the presence of CD8 T cells is not sufficient to induce T cell activation^16^, and the presence of an APC is required. A similar observation was made in the present study, as stimulation of RSV-specific T cells with RSV-peptide engineered exosomes did not induce ex vivo T cell activation without the addition of an APC (data not shown). Thery et al*.* have shown that exosomes can transfer functional pMHC-II complexes to DCs to activate antigen-specific CD4+ T cells^12^. Thus, it seems likely that a similar transfer of pMHC-I complexes occurs in our co-culture system with CD8+ T cells. Thery et al*.* also demonstrated that high costimulatory molecule expression, specifically CD80 and CD86, on the APC was required to mediate efficient exosome-mediated antigen presentation and T cell activation^12^. In our co-culture system, the addition of LPS to the exosome:T cell:DC co-cultures resulted in significantly increased antigen-specific IFN-γ production, supporting the requirement for matured CD11c + DCs to induce efficient antigen-presentation and T cell activation. Exosomes have similar properties or characteristics of their parental cells^24,25^. Given that LPS-stimulation induces costimulatory molecule expression in parental cells, peptide-engineered exosomes isolated from mature DCs could be expected carry both pMHC-I complexes and costimulatory molecules. Therefore, we initially hypothesized that exosomes prepared from LPS-matured APCs would be more immunogenic than those from immature APC, potentially negating the need for an additional external stimuli to activate the APC in the co-cultures. However, we observed no differences in the quantity of antigen-specific IFN-γ elicited from the T cells that were stimulated with exosomes from naïve DCs versus LPS-matured DCs. Thus, from our data, the presence of mature host APC to present exosome-derived pMHC complexes is the most important factor regulating the ex vivo response, rather than the activation state of the exosome-producing host cell.

Given their immunogenicity ex vivo, we expected that surface-engineered exosomes would have the capacity prime in vivo CTL responses. However, vaccination with peptide-engineered exosomes failed to elicit antigen-specific CD8 T cell responses in the animal, even when a prime-boost regimen was used. Andre et al*.* made a similar observation using DC-derived exosomes pulsed with a melanoma-derived CTL epitope^26^. In their work, inoculation with as high as 10^11^ peptide-pulsed exosomes failed to elicit an antigen-specific CD8 T cell response. As we observed in Fig. 1G and H, our engineered exosomes carry robust quantities of pMHC complexes, thus the dose that we administered offers sufficient antigen quantity for T cell priming. However, free exosomes have been estimated to be as much as 20-fold less efficient at stimulating T cell responses than their parent APC^27^. This weak stimulatory ability may be due to small size and wide vesicle dispersion^28^. In vivo, it seems likely that injected exosomes may be internalized by surrounding cells, thus diluting the availability of pMHC complexes for antigen-presentation. In vitro*,* we demonstrate that engineered-exosomes require the presence of an APC for efficient T cell activation. Others have shown that the inclusion of an APC in vivo similarly enhances the T cell priming capacity of exosomes. As an example, Andre et al*.* determined that loading of exosomes onto LPS-matured DC prior to injection significantly increased the capacity for CD8 T cell activation and differentiation *in vivo*^26^. Interestingly, however, Chaput et al*.* showed that the necessity for DC-loading of the exosomes could be overcome by the appropriate adjuvant. Mixing peptide-loaded exosomes with the TLR9 agonist, CpG ODN immediately prior to injection in the footpad enabled the exosomes to activate naïve, CD8 T cells in vivo^29^. Quil A was selected as an adjuvant in our studies. Quil A is a widely used saponin adjuvant that is known to stimulate balanced cellular and humoral immune responses, and to induce the generation of cytotoxic CD8 T cell responses^30^. It was our expectation that Quil A, like CpG, could enhance the immunogenicity of the peptide engineered exosomes in our system. However, inclusion of Quil A had no effects in vivo. Consistent with our results, Chaput et al*.* evaluated several other conventional adjuvants, including Montanide (ISA 720), which also failed to elicit exosome-induced CD8 T cell responses in vivo^29^. Only agonists for TLR3 (poly(I:C)) and TLR9 appeared to substitute for exosome-loaded DC in this system^29^. The threshold for activation of naïve CD8 T cells is known to be much higher than that of effector or memory CD8 T cells, thus further optimization may be required to overcome the naïve T cell activation threshold with peptide-engineered exosomes^31–34^. Additional factors such as cytokine environment and TLR crosslinking have been shown to reduce the threshold for naïve CD8 T cell activation^31–33^. This observation is consistent with that of Chaput et al., showing the importance of pattern recognition receptor engagement^29^. Recently the importance of robust innate immune activation on the induction of potent and long-lived memory T cell responses has also come to light^35^. In addition to their effect on adaptive immune cells, many prime-boost regimens have been shown to activate more efficient innate immune cells or engage innate immune memory, leading to more robust innate-adaptive immune cell cross-talk. In the current approach, we did not utilize an adjuvant or stimulant that would be expected to prime or boost innate immune memory, thus possibly leading to suboptimal efficacy of our peptide-engineered exosome vaccine. Our own future studies will focus on the incorporation of CpG or other potential TLR agonists that could be administered with the engineered exosomes to induce optimum innate immune activation and naïve T cell activation.

In addition to the consideration of additional adjuvants or innate immune stimulants, our future studies will further select the optimum antigenic payload for use in an engineered-exosome vaccine. The peptides selected for this study are well described, immunodominant CD8 T cell epitopes from RSV, and hence clear targets for vaccine development. However, peptides alone are not highly immunogenic. Although the strategic use of adjuvant may be sufficient to overcome this limitation, use of a larger protein fragment from RSV may prove more effective. Naslund et al*.* demonstrated that pMHC-I-loaded exosomes were poorly immunogenic**,** but loading the exosomes with a full-length protein elicited strong CD8 T cell responses in vivo^36^. The authors also demonstrated that CD4 T cell help was required for induction of exosome-induced CD8 T cell responses. In CD4 T cell deficient animals, even exosomes loaded with full-length protein failed to activate CD8 T cell responses. Naslund et al*.* also showed that B cells were required for processing the exosome-loaded proteins for CD8 and CD4 T cell activation^36^. Thus, induction of a robust immune response by exosomes likely involves complex interactions between multiple cell types. Loading exosomes with a larger protein fragment from RSV, coupled with an appropriate TLR agonist, may be the best approach for optimizing an engineered-exosome vaccine for prevention of a viral disease such as RSV.

In conclusion, we demonstrate that surface engineered exosomes successfully activated RSV-specific CD8 T cells ex vivo; however, injection of M_187–195_ or NS1_61–75_ engineered exosomes failed to prime virus-specific CD8 T cell responses in vivo. Importantly, we did not observe any adverse reactions after in vivo injection of surface-engineered exosomes. Further research is required to optimize the use of surface-engineered exosomes as a vaccine platform for use against viral diseases such as RSV.

## Methods

### Exosome isolation, therapeutic peptide surface engineering, and characterization

The proprietary fabrication of ExoRelease immunomagnetic nanographene particles produces flower-like nano pom-poms morphology with photo click chemistry, which has been licensed by Clara Biotech Inc. The MHC binding peptide M with sequence NAITNAKII (RSV M_187–195_) and NS1 peptide with sequence ICPNNNIVV (RSV NS1_61–75_) were synthesized by GenScript, confirming > 95% purity by high performance liquid chromatography (HPLC). These peptides conjugated onto ExoRelease particles respectively via NHS chemistry for affinity capture of MHC-I positive exosomes. The exosome isolation and surface engineering procedures were performed by incubating 100 mL of MHC binding peptide ExoRelease particles with 6 mL of cell culture medium at 4 °C overnight and shielded from light for MHC-I positive exosome capture and isolation. The solution tubes were placed onto the magnet for 1 min, then supernatant was discarded. Afterward, the particles were resuspended into 200 mL of 1 × phosphate buffered saline (PBS) buffer. Next, 15 mL of a solution containing β2-microglobulin (20 μg/mL) and peptide (100 μg/mL, either M peptide or NS1 peptide) for surface binding to form pMHC-I complex, was placed in a glass vial to incubate with rotation for 1 h at room temperature. The ExoRelease particles were separated from the reaction mixture by placing close to a magnetic bar for 1 min and discarding the supernatant; then the ExoRelease particles with surface engineered exosomes were washed three times with 1 × cool PBS buffer. Subsequently, exosomes were released from particles through photo-cleavage using a LED UV head at 365 nm wavelength exposed for 15 min (~ 6 mW/cm^2^). The conjugated PEG photo-cleavable linker with NHS moiety on bead surface allows the bond cleavage under light exposure to release intact, engineered peptide exosomes on demand, which further ensures the specificity for harvesting MHC-peptide modified exosomes. Harvested exosomes were characterized using the nanoparticle tracking analysis (NTA, Nano-Sight LM10, Malvern Panalytical) to determine size distribution and particle concentration of engineered exosomes. NTA post-acquisition parameters were adjusted to a screen gain of 10.0 and a detection threshold to 5. Standard 100 nm nanoparticles were used for calibration. Samples were diluted in 1 × PBS before every measurement and repeated for five times. The zeta potential was measured using ZetaVeiw (Particle Metrix).

NanoView microarray for characterization of exosome MHC-I expression level was conducted per standard protocols provided by NanoView Biosciences (Brighton, MA). Processed JAWS cell culture media was diluted appropriately using the EV binding buffer (solution A, pH 7.4), then 35 µL of diluted sample was dropped on microarray chips for incubation overnight. Each three chip spots were pre-coated with capture antibodies CD81 (clone Eat-2, mouse, BioLegend) and CD9 (clone MZ3, mouse, BioLegend), and negative controls HIgG (clone HTK888, mouse, BioLegend). Microarray chips were washed four times with solution A at 150 rpm/min, 3 min for each time. After washing, 300 µL blocking solution was incubated with each chip for 1 h at room temperature and protected from light, which contains two detection antibodies including 0.6 µL Alexa Fluor 647-conjugated CD63 (clone NVG-2, mouse, BioLegend) and 2.5 µL PE-conjugated MHC class I (clone AF6-88.5.5.3, mouse, Invitrogen). Subsequently, microarray chips were washed again with solution A, solution B and distilled water, then chips were kept air dry for imaging by ExoView R100 (NanoView Biosciences) equipped with 40 × objective lens (Olympus, Japan). Data was analyzed and quantified using off-line ExoViewer3 EAP_v3 software.

The bead-based flow cytometry analysis (CytoFlex, Beckman) was performed to characterize our ExoRelease particle captured exosomes and their surface engineering with RSV peptides. The exosomes were labeled by PKH67 (Sigma-Aldrich) per vendor’s instruction. The RSV peptides (M and NS) were labeled by Alexa Fluor 555 (Thermo Fisher).

### Characterization of engineered exosomes using SEM and TEM imaging

For scanning electron microscope observation, exosome bound particles were resuspended in 200 μL PBS solution and washed 2 times with pure water. 5 μL exosome bound particle solution was added to clean silicon chips and immobilized by drying under a ventilation hood. Samples on silicon chips were mounted on a SEM stage by carbon paste with 30 s sputtering coating. SEM imaging was performed under low beam energies (7 kV) with 10 nA on Hitachi SU8230 field emission scanning electron microscope.

For TEM imaging, ~ 5 μL of harvested exosomes were dropped onto formvar carbon coated copper Grid 200 mesh (Electron Microscopy Sciences) for 5 min followed by 3 min of negative staining with 2% aqueous uranyl acetate. Excess liquid was blotted by a filter paper. Total grid preparation was performed at room temperature until completely air-dried under a ventilation hood for 25 min. The imaging was performed immediately after fixation using the Tecnai G2 Spirit TWIN Transmission Electron Microscope at 120 kV.

### RSV isolation

Human epithelial type 2 (HEp-2) cells (ATCC, CCL-23) were maintained in complete minimal essential media (cMEM), composed of MEM medium (Gibco) containing 10% fetal bovine serum (FBS, Gibco), 2 mM l-glutamine (Gibco), 1% antibiotic antimycotic (Gibco), and 1 mM sodium pyruvate (Gibco) until they are approximately 80 to 90% confluent. RSV strain A2 (ATCC, VR-1540) was inoculated into the HEp-2 cells with serum-free MEM. After 2 h of virus inoculation, the supernatant was removed, and cMEM was added. For storage, RSV infected HEp-2 cells were frozen at − 80 °C from 48 to 72 h after infection. Virus-infected HEp-2 cells were thawed, and cultures were centrifuged at 3200×*g* for 15 min at 4 °C to remove cell debris. Virus supernatants were collected and added to the polyethylene glycol 8000 in NT buffer containing 150 mM NaCl and 50 mM Tris–HCl (10% w/v). The supernatants were incubated at 4 °C for an hour with stirring and then centrifuged to precipitate the RSV at 3200×*g* for 40 min at 4 °C. The virus was suspended in 3% sucrose Dulbecco's Modified Eagle Medium (DMEM, Gibco) and stored at − 80 °C. HEp-2 cells were inoculated with tenfold serial dilutions of RSV in a 96 well flat bottom plate. The titration was measured by cytopathic effects. The Reed and Muench calculation was used to get the virus titer.

### Animal study and ex vivo experiments

All mice procedures were in accordance with the relevant guidelines and regulations, and experimental protocols were approved by the Iowa State University Institutional Animal Care and Use Committee (IACUC) 18-074. Additionally, all animal experiments were conducted in compliance with the ARRIVE (Animal Research: Reporting of In Vivo Experiments) guidelines. Six to seven week old C57BL/6J female mice were purchased from Jackson Laboratories. Mice were challenged intranasally with RSV. Animals were anesthetized with isoflurane prior to intranasal inoculation with 10^7^ PFU RSV in 60 μL 3% sucrose in DMEM and were sacrificed on day 8 post-infection by carbon dioxide (CO_2_) asphyxiation, and then the lung tissues were harvested. Five RSV infected C57BL/6 mice were euthanized and spleens and lungs were collected for CD8+ T cells. Another five non-infected C57BL/6 (naïve) mice were euthanized and spleens were collected for CD11c + cells. Magnetic activated cell sorting (MACS) was used to sort CD8+ T cells and CD11c+ DCs according to the manufacturer's instructions (Miltenyi Biotec). The exosome:T cell:DC co-culture model was adapted from previously described methods^16^. CD8+ T cells and CD11c+ DC cells (5:3 ratio) were incubated for 72 h with escalating concentration (5 μL, 10 μL, 25 μL) of exosomes engineered with M_187–195_ or NS1_61–75_ peptides with or without LPS (100 ng/mL, InvivoGen) . Cells were cultured in complete RPMI (cRPMI), composed of RPMI 1640 medium (Gibco) containing 10% FBS, 2 mM l-glutamine, 1% antibiotic antimycotic, and 1 mM sodium pyruvate. cRPMI was used as a negative control, and M peptide (5 μg/mL, GenScript), NS1 peptide (5 μg/mL, GenScript), and ConA (5 μg/mL, MP Biomedicals) were used as positive controls.

### Exosome immunization for challenging RSV infected mouse model

Five mice per group were immunized (prime-boost) subcutaneously with 100 μL (1.7 × 10^9^ particles/mL) of engineered exosomes harboring either M or NS1 peptides on their surface with or without Quil A adjuvants (10 μg/mouse). There were 2 weeks gap between prime and boost vaccination. Mice were challenged intranasally with RSV, 2 weeks after boost. The same protocols as ex vivo experiments were used to inoculate the virus and sacrifice the mice. They were anesthetized with isoflurane prior to intranasal inoculation with 10^7^ PFU of RSV in 60 μL 3% sucrose in DMEM. Mice were sacrificed on day 3 or 6 post-infection by carbon dioxide (CO_2_) asphyxiation and lung tissues were harvested (same as ex vivo). Lung cells were stimulated and incubated with cRPMI media, M peptide (5 μg/mL), and NS1 peptide (5 μg/mL). Positive controls used were PMA (50 ng/mL, Sigma-Aldrich) and Ionomycin (1 μg/mL, MP Biomedicals) for flow cytometry and ConA (5 μg/mL) for ELISA.

### Lung cell isolation

The lungs were diced and digested by collagenase type I (3 mg/mL, Gibco) in cRPMI for 30 min at 37 °C. After incubation, the tissues were processed through a 70 μm strainers to prepare a single cell suspension. The cell suspension was washed twice with cRPMI at 1500 rpm for 5 min at 4 °C.

### Tetramer and intracellular cytokine staining (ICS)

After 12-h incubation with stimuli followed by 6-h incubation with Brefeldin A (Becton Dickinson Biosciences) for all wells and PMA/Ionomycin only for positive controls, virus-specific T cells were detected with tetramers of H-2D^b^ RSV M_187–195_ peptide. Cells were stained with fluorochrome-conjugated antibodies against FITC anti-mouse CD45 (clone 30-F11, mouse, eBioscience,), PerCP/Cyanine5.5 anti-mouse CD8a (clone 53–6.7, mouse, BioLegend,), APC H-2D^b^ RSV M_187–195_ tetramer (MBL), and aqua live/dead fixable dead cell stain (Invitrogen) for 30 min at 4 °C. Cells were fixed and permeabilized according to the manufacturer's instructions (Becton Dickinson Biosciences) and stained with APC/Cyanine7 fluorochrome-conjugated anti-mouse IFN-γ antibodies (clone XMG1.2, mouse, BioLegend) for 30 min at room temperature. After staining, cells were washed and analyzed by flow cytometry. Cells were analyzed on a Becton Dickinson FACSCanto. Data were analyzed by using FlowJo software version 10.7.1 (FlowJo, LLC). A representative gating strategy for analysis of tetramer and intracellular cytokine staining is included in Supplementary Fig. 1. 

### ELISAs

Mouse IFN-γ DuoSet ELISA Development kit was purchased from R&D systems and ELISAs were performed according to manufacturer’s instructions. Sandwich ELISAs were used to quantify mouse IFN-γ in cell culture supernatants. As previously described, lung cells were stimulated and incubated with cRPMI media, M peptide (5 μg/mL), NS1 peptide (5 μg/mL), and ConA (5 μg/mL). The supernatants were collected after 72-h or 5-day stimulation with those stimuli. The supernatant samples were diluted 1:4 with reagent diluent buffer. All samples and standards were plated in duplicates. The results of ELISA are representative of three independent ex vivo experiments and that of one in vivo experiment.

### Statistics

Results were shown as averages ± standard errors of the mean (SEM). Statistical analysis was determined by one-way and two-way Analysis of Variance (ANOVA) using Prism software version 9.0.0 (GraphPad Software, LLC).

## Supplementary Information

Supplementary Information.
