# Supplementary material for: Development of surface engineered antigenic exosomes as vaccines for respiratory syncytial virus
Source: Sci Rep. 2021 Nov 1;11:21358. doi: 10.1038/s41598-021-00765-x (PMC8560785; doi:10.1038/s41598-021-00765-x)
Supplement: Supplementary file 1 — Supplementary Information. [file 41598_2021_765_MOESM1_ESM.pdf]

Development of surface engineered antigenic exosomes as vaccines for respiratory syncytial virus

Suyeon Hong[1], Shaobo Ruan[2], Zachary Greenberg[2], Mei He[2] and Jodi L. McGill[1]

[1]Department of Veterinary Microbiology and Preventive Medicine, Iowa State University, Ames, IA

[2]Department of Pharmaceutics, College of Pharmacy, University of Florida, Gainesville, FL

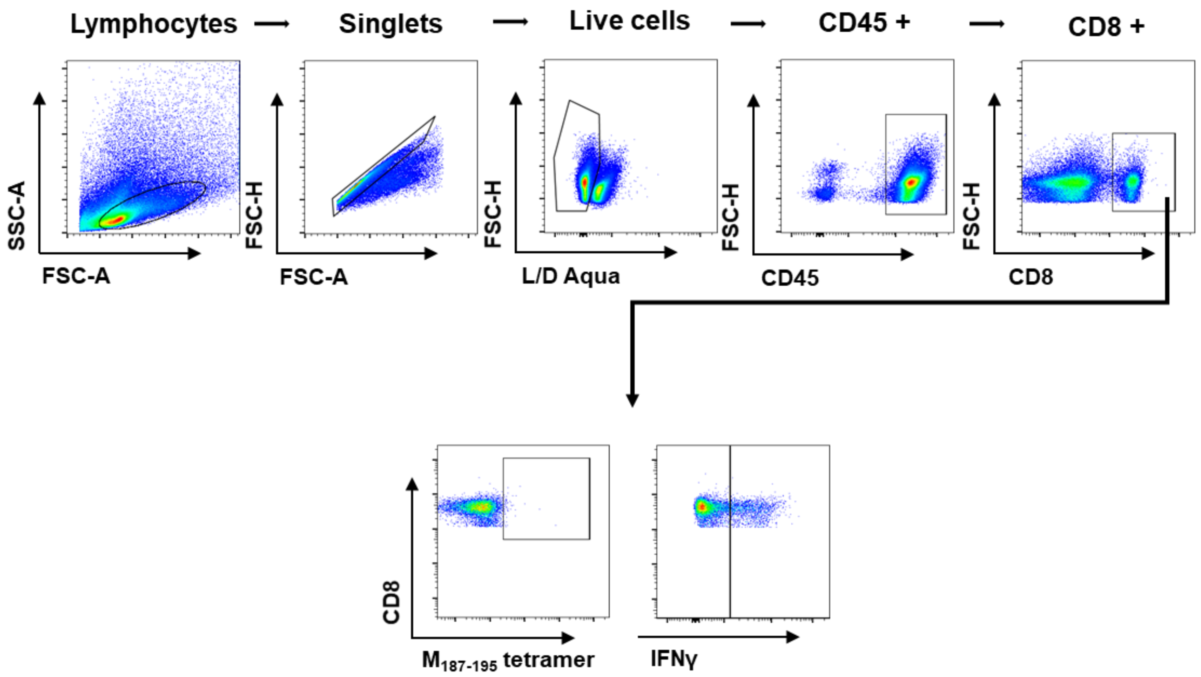

Supplementary Fig. 1. Gating strategy of H-2D^{b} RSV M$_{187–195}$ tetramer staining and intracellular cytokine staining. Lymphocytes were gated using forward and side scatter, and single cells were gated by forward scatter height and area. After gating live cells, CD45-positive and CD8-positive cells were gated. The gate was set on CD8+ cells that are positive for M$_{187–195}$ tetramer (lower left) and positive for IFN-γ (lower right).
